# Risk of graft loss in kidney transplant recipients after aortic valve replacement

**DOI:** 10.17305/bjbms.2022.7720

**Published:** 2023-01-06

**Authors:** Stefan Büttner, Carolin Zöller, Sammy Patyna, Anisa Gradascevic, Helge Weiler, Mark Rosenberg, Thomas Walther, Andreas M Zeiher, Helmut Geiger, Mariuca Vasa-Nicotera, Ingeborg A Hauser, Stephan Fichtlscherer

**Affiliations:** 1 Medical Clinic III – Department of Nephrology, University Hospital Frankfurt, Frankfurt am Main, Germany; 2 Medical Clinic I – Cardiology, Pneumology, Nephrology and Intensive Care Medicine, Klinikum Aschaffenburg-Alzenau, Aschaffenburg, Germany; 3 Medical Clinic II – Nephrology, Agaplesion Markus Krankenhaus, Frankfurt am Main, Germany; 4 Medical Clinic III – Department of Cardiology, University Hospital Frankfurt, Frankfurt am Main, Germany; 5 Department of Thoracic and Cardiovascular Surgery, University Hospital Frankfurt, Frankfurt am Main, Germany

**Keywords:** Aortic valve stenosis (AS), aortic valve replacement, transcatheter aortic valve implantation (TAVI), kidney transplant recipients (KTR), graft survival.

## Abstract

Surgical aortic valve replacement (SAVR) in kidney transplant recipients (KTR) is associated with high morbidity and mortality, and an increased risk of postoperative graft failure potentially leading to graft loss. Transcatheter aortic valve implantation (TAVI) emerged as an alternative in high-risk patients. However, data on TAVI in KTR are limited. We performed a retrospective analysis of 40 KTR in which aortic valve replacement was performed at our center between 2005 and 2015. The outcomes and follow-up of TAVI (*n* = 20; 2010-2015) and SAVR (*n* = 20; 2005–2015) were analyzed with respect to patient and graft survival. Baseline characteristics in both groups were comparable. Hospital stay after TAVI was significantly shorter compared to SAVR (19 [11.5–21.75] days vs. 33 [21–62] days, *p* = 0.001). Acute graft failure occurred more frequently after SAVR (45% vs. 89.5%; *p* = 0.006). Thirty-day mortality was 10% in both groups. However, in-hospital mortality reached 25% in the SAVR group (TAVI 10%), indicating a more complicated course after surgery. Moreover, during a median follow-up time of 1928 days in TAVI patients and 2717 days in patients after SAVR, graft loss occurred only in the surgically treated group (*n* = 7). While one-year survival after TAVR was 90% compared to 69% after SAVR, long-term follow-up showed comparable results (at 5 years: TAVI 58% vs. 52% SAVR; log-rank-test: *p* = 0.86). In KTR, TAVI can be performed with good mid- to long-term results. Compared to SAVR, renal outcomes seem to be improved after TAVI, suggesting better graft survival.

## Introduction

Aortic valve stenosis (AS) is one of the most common valve diseases in patients with chronic kidney disease [1], especially with end-stage renal disease (ESRD) [1, 2]. Compared to the general population, the prevalence of heart valve diseases in ESRD patients is higher with about 13% of patients suffering from AS after four years of dialysis [2, 3]. Data on the incidence of AS after kidney transplantation are scarce, but the risk for developing AS in kidney transplant recipients (KTR) is likely to increase with longer time on dialysis due to increasing waiting time, improved survival, and increasing age. In the general population, surgical aortic valve replacement (SAVR) is the gold standard therapy for AS with overall low perioperative mortality [4]. However, data regarding SAVR in KTR are limited showing an increased morbidity and mortality [5], with acceptable long-term survival rates of about 50% after five years [5]. Large, randomized trials have been performed comparing transcatheter aortic valve implantation (TAVI) to SAVR for high- [6] and intermediate-risk patients [7] showing non-inferiority of TAVI. TAVI also emerged as a viable option for dialysis patients [8, 9]. However, KTR represent a special population due to impaired renal function, ongoing immunosuppressive therapy (e.g., steroids, mycophenolate, and calcineurin inhibitors) with a higher risk for infections and wound healing deficits [10], and a high burden of comorbidities. Similar to SAVR [11], the outcome of TAVI in general population is determined by kidney function prior to the intervention [12–15] and acute kidney injury (AKI) following TAVI [16, 17]. To date, data on TAVI in KTR with a functioning kidney graft are limited [18–20]. The aim of this study was to investigate short- and long-term data of KTR undergoing TAVI or SAVR, and analyze the outcomes with respect to patient and graft survival.

## Materials and methods

### Patients

All KTR with a functioning graft and a high-grade AS undergoing either TAVI or SAVR at University Hospital Frankfurt between 2005 and 2015 were included in this retrospective cohort study. The University Hospital Frankfurt is a tertiary care institution including a kidney transplant center which performs around 70 kidney transplantations per year. During the study period, 747 kidney transplantations were performed. The study was conducted in accordance with the 1975 Declaration of Helsinki and was approved by the institutional review board (file number 429/15) of the Goethe University.

Treatment strategy was discussed for every individual case within the local interdisciplinary heart team. Demographic, clinical, laboratory, echocardiographic, and treatment parameters were systematically retrieved from the medical case files for every patient. For all patients we calculated the logistic EuroSCORE and the EuroSCORE II using the calculator on http://www.EuroSCORE.org/calc.html, as well as the Society of Thoracic Surgeons’ (STS) risk model using the calculator on http://riskcalc.sts.org/stswebriskcalc/#/calculate. Follow-up of all patients took place at the University hospital or in the associated outpatient clinic (“Kuratorium für Dialyse und Nierentransplantation e.V. Schleusenweg,” Frankfurt am Main, Germany).

### Definitions and clinical endpoints

Baseline kidney transplant function was retrieved from previous consultations (at least three months prior to admission). Postoperative incidence of acute graft failure was compared using The Acute Kidney Injury Network (AKIN) classification [21]. Procedure-related complications were assessed following the Valve Academic Research Consortium (VARC) 2 definition [22].

Perioperative preparation followed a standardized protocol in both groups. SAVR and TAVI were performed by a highly experienced heart-team. By protocol, in both groups, antibiotic prophylaxis was given prior to surgery or intervention. Furthermore, a continuous infusion of hydrocortisone (200 mg/day) was administered during and after the surgery, replacing immunosuppressive therapy. Nephroprotection was performed by administration of intravenous fluid administration until euvolemia and discontinuing nephrotoxic medications prior to the intervention. Hemodialysis, if necessary, was performed as either intermittent or continuous hemodialysis/hemodiafiltration depending on the hemodynamic status of the individual patient. Anticoagulation of the dialysis circuit was performed with heparin or regional citrate anticoagulation.

### Statistical analysis

Continuous variables are shown as median and interquartile range (IQR). Categorical variables are reported as frequencies and percentages. Continuous and categorical variables were compared using Mann–Whitney U test, Wilcoxon matched pair test, and Fisher’s exact test. To analyze the patient and graft survival, we used Kaplan–Meier estimator. All *p*-values reported are two-sided. Statistical significance was assumed when the *p*-value was <0.05. Statistical analyses were performed using BiAS (http://www.bias-online.de) and Prism 5 (GraphPad Software Inc; San Diego, CA, USA).

## Results

### Patient characteristics

The study population comprised of a total of 40 KTR after aortic valve replacement, including 20 patients after SAVR and 20 patients after TAVI. As shown in Table 1, clinical (including gender, comorbidities, BMI, and dialysis time prior to transplantation) and echocardiographic data, as well as maintenance immunosuppressive therapy at admission (Table 2) were comparable between the groups. Patients in the TAVI group tended to be older (69 vs. 65.5 years; *p* = 0.06). Importantly, perioperative risk assessment of both groups using the STS-Score (5.27 vs. 5.19; *p* = 0.74) and EuroScore II (4.53 vs. 4.89; *p* = 0.78) showed no difference between the two groups. Three patients (15%) underwent interventional coronary angioplasty for significant coronary artery stenosis prior to TAVI, within one week of the index procedure. In the SAVR group, aortic valve replacement was performed as isolated procedure in 9 or combined with coronary artery bypass grafting in 11 patients.

**Table 1:** Patient characteristics of study population

**Values**	**TAVI**		**SAVR**		***p*-value**	**Combined**		**Isolated**	
	***n* = 20**		***n* = 20**			***n* = 11**		***n* = 9**	
**Epidemiology**									
Age, years	69	(67–74)	65.5	(59.9–68)	0.06	67	(62–69)	65	(57.5–67.5)
Gender, male	14	(70)	14	(70)	1.00	7	(64)	7	(78)
BMI, kg/m^2^	27.5	(24.2–31.5)	26.8	(22.1–31.8)	0.38	24.3	(21.8–26.9)	31.8	(23–33.9)
Time since Tx, months	73.5	(43.3–130.5)	95	(23–243.8)	0.61	83	(20–132)	150	(24–277)
Time on dialysis prior Tx, months	59	(24.2–31.5)	60.0	(23–107)	0.61	72	(42–107)	43	(17–118.8)
**Risk calculation**									
Euro SCORE II	4.53	(2.9–8.2)	4.89	(2.8–9.4)	0.78	7.94	(4.77–10.48)	2.77	(2.05–5.39)
STS Score	5.27	(3.7–6.1)	5.19	(2.7–7.7)	0.74	5.463	(3.64–8.72)	3.34	(2.24–6.52)
**Comorbidities**									
Diabetes mellitus	13	(65)	9	(45)	0.34	5	(45)	4	(44)
COPD	7	(35)	10	(45)	0.52	6	(55)	4	(44)
Previous myocardial infarction	2	(10)	6	(30)	0.24	5	(45)	1	(11)
Atrial fibrillation	6	(30)	11	(55)	0.20	6	(55)	4	(44)
Peripheral artery disease	10	(50)	4	(21)	0.10	0		4	(44)
Cerebrovascular disease	3	(15)	3	(15)	1.00	0		3	(35)
Previous pace maker	1	(5)	4	(20)	0.34	3	(27)	1	(11)
Previous CABG	4	(20)	0	(0)	0.11	0		0	
Coronary artery disease	13	(65)	12	(60)	1.00	10	(91)	2	(22)
Hypertension	20	(100)	19	(95)	1.00	10	(91)	8	(89)
**Echocardiographic data**									
LV-EF, %	65	(56–65)	60	(55–65)	0.51	60	(42.3–65)	65	(55–65)
Aortic valve area, cm^2^	0.8	(0.7–1.0)	0.8	(0.7–0.9)	0.12	0.79	(0.7–0.825)	0.6	(0.6–0.8)
Mean transaortic pressure gradient, mmHg	44.5	(35.5–50)	52	(38.5–62.5)	0.19	46	(35.5–51.5)	61.5	(55.75–67.25)
Max transaortic pressure gradient, mmHg	71	(61.3–77.8)	83	(65–93.5)	0.09	80	(61.5–83.5)	90.5	(81.75–103.3)
IVS, mm	15	(14–16)	16	(15–16)	0.21	15	(14.25–16)	16	(15.17.5)
LA diameter, mm	46	(42.5–49.5)	45	(41.8–52.8)	0.95	42.5	(37.5–50)	45	(44–53)
LVEDP, mmHg	49	(42.8–52.3)	51.5	(45.5–58.3)	0.22	53	(43.5–58.75)	51	(45.5–56.5)
LVPW, mm	14	(13.5–15)	15	(13–15.3)	0.92	14.5	(13–15)	15	(13.5–16)
PAP, mmHg	41	(37–47)	36	(30–55)	0.59	43.5	(35.25–57.5)	30	(26.5–49.5)

Data presented as numbers (percentage) or median (interquartile range). BMI: Body mass index; COPD: Chronic obstructive pulmonary disease; CABG: Coronary artery bypass graft; LV-EF: Left ventricular ejection fraction; IVS: Intraventricular septum; LA: Left atrium; LVEDP: Left ventricular end-diastolic pressure; LVPW: Left ventricular posterior wall; PAP: Pulmonal artery pressure; Tx: Kidney transplantation; TAVI: Transcatheter aortic valve implantation; SAVR: Surgical aortic valve replacement; STS: Society of Thoracic Surgeons.

**Table 2:** Maintenance immunosuppressive therapy on admission

**Immunosuppressive therapy**	**TAVI**		**SAVR**		***p*-value**
	***n* = 20**		***n* = 20**		
CNI mono			1	5	1.000
CNI+steroid	10	50	5	25	0.191
CNI+MMF/MPA	1	5	3	15	0.604
CNI+Aza	3	15	1	5	0.604
CNI+MMF/MPA+steroid	4	20	2	10	0.661
MMF/MPA+steroid	2	10	8	40	0.065

Data presented as numbers (percentage). CNI: Calcineruin inhibitor; MMF: Mycophenolat mofetil; MPA: Mycophenolic acid; Aza: Azathioprine; TAVI: Transcatheter aortic valve implantation; SAVR: Surgical aortic valve replacement.

### Procedure data and in-hospital course

Procedure data and hospital course are given in Table 3. Transfemoral route was used in 90% of TAVI procedures. In two patients, the transapical access was used. Different types of valves were implanted (13 Edwards Sapien, 6 CoreValve, and 1 Portico). Median length of fluoroscopy time during TAVI was 24 min (IQR: 19.25–33.5) and a median amount of 138 ml (102.5–177.5) of contrast dye was used during the intervention. Of the surgically treated patients, 17 received bioprosthetic valves (14 Edwards Perimount, 2 Medtronic Mosaic, and one SJM Toronto) and 3 patients received mechanical valves (2 Medtronic Advantage and one Edwards MIRA 3600). Median time of surgery in the cohort was 261 min (195.5–311) with shorter duration of isolated SAVR [201 min (172–257)] compared to the combined surgery [310 min (270–360)]. Median time on extracorporeal circulation was 139 min (106.75–176.75) with shorter time on extracorporeal circulation for patients with isolated SAVR [115 min (68.5–125)] compared to patients with combined surgery [171 min (149.3–191)]. Median aortic cross-clamping time was 104 min (67.25–121.25) (isolated SAVR: 75.5 min [45.5–98]; combined surgery: 121 min [113.5–129.8]).

**Table 3:** Procedure data and in-hospital course

**Values**	**TAVI**		**SAVR**		***p*-value**	**Combined**		**Isolated**	
	***n* = 20**		***n* = 20**			***n* = 11**		***n* = 9**	
**Procedure data**									
Length of fluoroscopy time, min	24	(19.3–33.5)	n/a			n/a		n/a	
Contrast dye used, ml	138	(102.5–177.5)	n/a			n/a		n/a	
Transfemoral route	18	(90)	n/a			n/a		n/a	
Operation time, min	n/a		261.0	(195.5–311)		310	(270–360)	201	(172–257)
Extracorporeal circulation, min	n/a		139.0	(106.8–176.8)		171	(149.3–191)	115	(68.5–125)
Aortic cross clamp time, min	n/a		104.0	(67.3–121.3)		121	(113.5–129.8)	75.5	(45.5–98)
**In-hospital course**									
Length of hospital stay, days	19	(11.5–21.8)	33	(21–62)	**0.002**	27.5	(17.25–61.25)	42	(25.5–104.5)
Length of ICU stay, days	4.5	(3–6.8)	8	(3–24)	0.11	10	(2,75–29.5)	8	(3–24.5)
RBC transfusion	11	(55)	18	(90)	**0.03**	10	(91)	9	(100)
Platelet transfusion	3	(55)	9	(45)	0.08	4	(36)	5	(56)
FFP transfusion	4	(20)	13	(65)	0.01	8	(73)	6	(67)
S-creatinine on admission, mg/dl	1.9	(1.55–2.94)	2.04	(1.24–2.95)	0.68				
S-creatinine at 24 h, mg/dl	2.25	(1.33–2.95)	2.47	(1.7–3.12)	0.37				
CRP on admission, mg/dl	0.62	(0.09–1.34)	0.44	(0.24–2.15)	0.68				
CRP at 48 h, mg/dl	6.43	(1.64–8.57)	17.9	(11.68–23.01)	**0.000004**				

Data presented as numbers (percentage) or median (interquartile range). Bold values represent statistical significance. ICU: Intensive care unit; RBC: Red blood cell; FFP: Fresh frozen plasma; CRP: Creactive protein; TAVI: Transcatheter aortic valve implantation; SAVR: Surgical aortic valve replacement.

The median duration of the hospital stay in the TAVI group was 19 days (11.5–21.75). In contrast, patients after SAVR had a significantly longer median hospital stay with 33 days (21–62) (*p* = 0.002). The length of stay in intensive care unit (ICU) did not differ significantly between the two groups (TAVI: 4.5 days (3–6.8) vs SAVR: 8 (3–24); *p* = 0.11). Patients after SAVR needed significantly more blood transfusions (RBP) (TAVI: 55% vs SAVR: 86%, *p* = 0.03), as well as fresh frozen plasma (FFP) (TAVI: 20% vs SAVR: 65%; *p* = 0.01).

### Procedure-related complications

As shown in Table 4, the spectrum of complications differed between the two groups. After SAVR, 30% of cases required re-sternotomy, 40% of cases were complicated by wound healing deficits, and 55% of patients suffered from various infections (Table 5), some of which were severe (e.g., four cases of pneumonia and two cases of sepsis). In contrast, infectious complications were very rare in the TAVI group (5%, one case of pneumonia). In the group, 55% of the procedures were complicated by vascular complications, such as dissection, aneurysm, and bleeding at the access site. One TAVI patient was converted to open heart surgery after ventricle rupture during TAVI using the transapical access and one patient suffered from peri-interventional minor stroke without neurologic sequelae.

**Table 4:** Complications during hospital stay

**Complications**	**TAVI**		**SAVR**		***p*-value**	**Combined**		**Isolated**	
	***n* = 20**		***n* = 20**			***n* = 11**		***n* = 9**	
Infectious complications	1	(5)	11	(55)	**0.0012**	6	(55)	5	(56)
Major vascular complications	3	(15)							
Minor vascular complications	8	(40)							
Life-threatening bleeding^1^	2	(10)	7	(35)	0.1274	4	(36)	3	(33)
Major bleeding^2^	7	(35)	3	(15)	0.2733	2	(18)	1	(11)
Minor cerebrovascular accident	1	(5)							
Myocardial infarction			1	(5)				1	(11)
Wound healing deficits			8	(40)		6	(55)	2	(22)
Conversion to open heart surgery	1	(5)							
Re-sternotomy			6	(30)		3	(27)	3	(33)
Pacemaker implantation	3	(15)	1	(5)	0.605	1	(9)		

Data presented as numbers (percentage). ^1^Defined as bleeding with a drop in hemoglobin ≥5 g/dl; ^2^Defined as bleeding with a drop in hemoglobin ≥3 g/dl. TAVI: Transcatheter aortic valve implantation; SAVR: Surgical aortic valve replacement.

**Table 5:** Infectious complications and causes of death

**Complications/cause of death**	**TAVI**		**SAVR**	
	***n* = 20 **		***n* = 20**	
**Infectious complications during hospitalization**				
Pneumonia	1	(5)	4	(20)
Sepsis			2	(10)
Endocarditis			1	(5)
Cholangitis			1	(5)
Wound infection			2	(10)
Urinary tract infection			1	(5)
**Cause of death during hospitalization**				
Cardiac	2	(10)		
Infection			5	(25)
**Cause of death during follow-up**	*n* = 18		*n* = 15	
Infection	2	(11)	1	(6.7)
Cardiac	3	(16.7)	2	(13.3)
Malignancy	1	(5.5)		
Unknown			2	(13.3)

Data presented as numbers (percentage). TAVI: Transcatheter aortic valve implantation; SAVR: Surgical aortic valve replacement.

### Graft function and graft survival

**Figure 1.**
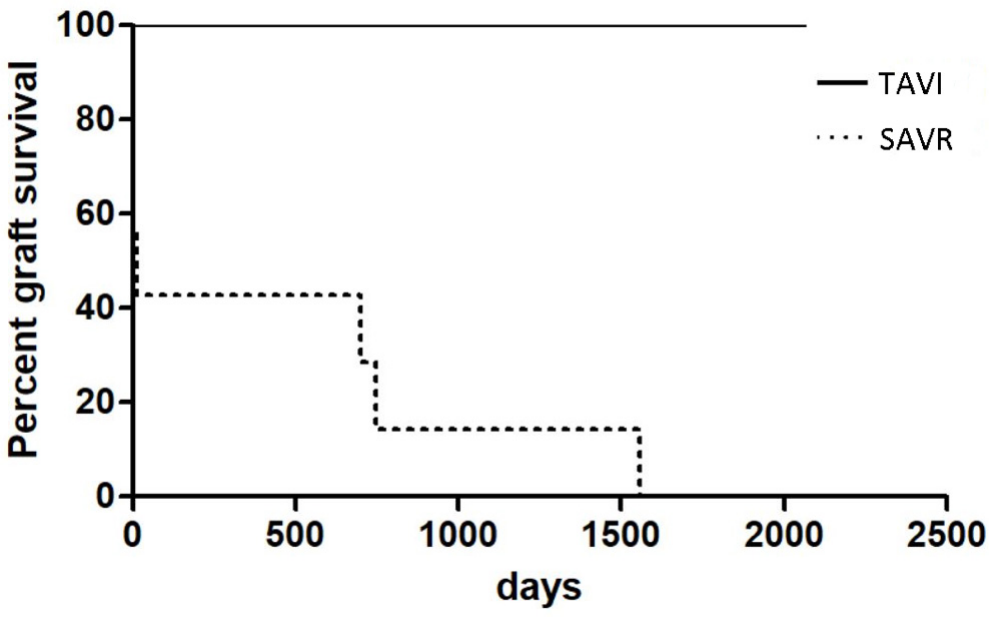
**Kaplan–Meier analysis for graft survival between the TAVI and SAVR group.** TAVI: Tanscatheter aortic valve implantation; SAVR: Surgical aortic valve replacement.

Compared to TAVI, acute graft failure was almost twice as frequent after SAVR (45% vs. 90%, *p* = 0.006). After the procedure, 25% of TAVI patients and 55% of patients after SAVR needed renal replacement therapy (*p* = 0.053). Detailed information is presented in Table 6. Interestingly, there was no difference in the incidence and severity of acute graft failure between the two subgroups of isolated or combined SAVR. While transplant function in the TAVI group returned to baseline in all patients suffering from acute graft failure, a total of four patients after SAVR remained on hemodialysis at discharge. In addition, three patients after SAVR developed graft loss during follow-up. Long-term graft survival after SAVR was reduced to 50% after five years (*p* = 0.0002). The median time to graft loss after SAVR was 7 days (1–746). Notably, there was no graft loss during hospital stay or follow-up in the TAVI group (Figure 1).

**Table 6:** Graft function

**Graft function**	**TAVI**		**SAVR**		***p*-value**	**Combined**		**Isolated**	
	***n* = 20**		***n* = 20**			***n* = 11**		***n* = 9**	
**Acute graft injury**	9	(45)	18	(90)	**0.006**	9	(82)	9	(100)
AKIN I	3	(15)	2	(10)	1.000	0		2	(22)
AKIN II	1	(5)	3	(15)	0.605	2	(18)	1	(11)
AKIN III	5	(25)	13	(65)	**0.024**	7	(64)	6	(67)
Need for dialysis	5	(25)	11	(55)	0.053	6	(55)	5	(56)
**Graft loss**	0		7	(35)		2	(18)	5	(56)
During hospital stay	0		4	(20)		1	(9)	3	(33)
During follow-up	0		3	(15)		1	(9)	2	(22)
Time to graft loss in days	n/a		10	(1–746)					

Data presented as numbers (percentage) or median (interquartile range). Bold values represent statistical significance. AKIN: Acute Kidney Injury Network; TAVI: Transcatheter aortic valve implantation; SAVR: Surgical aortic valve replacement.

### Patient outcomes

**Figure 2.**
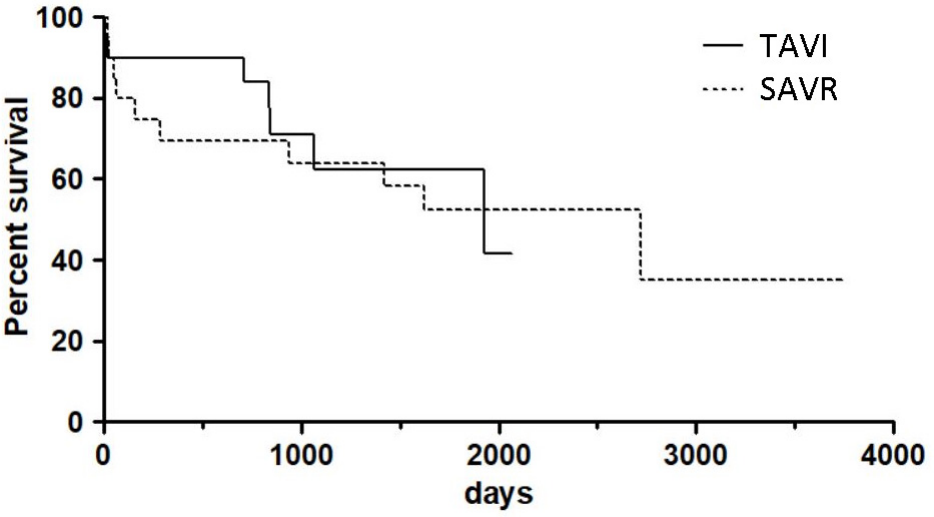
**Kaplan–Meier analysis for patient survival between the TAVI and SAVR group**. TAVI: Transcatheter aortic valve implantation; SAVR: Surgical aortic valve replacement.

Thirty-day mortality was 10% in both groups. However, the in-hospital mortality reached 25% in the SAVR group (TAVI 10%). Median follow-up time was 1928 days in TAVI patients and 2717 days in patients after SAVR. One-year survival estimated by Kaplan–Meier analysis was 90% after TAVI compared to 69% after SAVR. In the further long-term follow-up, a similar survival rate was achieved in both groups (TAVI 58% vs SAVR 52%, log-rank-test: *p* = 0.86) (Figure 2).

## Discussion

Our retrospective study investigating and following a cohort of KTR undergoing aortic valve replacement provides interesting information on patient and graft outcomes after surgical or interventional aortic valve replacement in this special population. TAVI seems to provide better short- to mid-term outcomes, mostly because of a reduced incidence of severe in-hospital complications. However, long-term results seemed to be comparable to SAVR. Most importantly, the rate of severe acute graft failure, and even graft loss, seems to be markedly reduced after TAVI compared to SAVR. This goes in line with a recent analysis from the U.S. Nationwide Representative Study showing a lower rate of AKI in KTR after TAVI compared to SAVR [23].

Data regarding outcomes after heart valve surgery in KTR are limited, suggesting an increased morbidity and mortality in these patients. Reported in-hospital mortality ranges from 5% to 18.7% [5, 24, 25] and reveals a high two-year mortality rate after valve surgery of 40% [5]. Due to its minimally invasive nature and lack of need for extracorporeal heart and lung support, TAVI may offer an improved outcome in the high-risk population of KTR. Data of TAVI in eight KTR from our center, showing a lower in-hospital and one-year mortality, support this assumption [18]. Al-Rashid et al. [19] reported low short-term mortality in eight KTR undergoing TAVI, while the mid-term mortality after 1 and 2 years reached 38% and 53%, respectively. Notably, both cohorts had a very high median length of hospital stay (19 and 33 days, respectively), indicating a prolonged and complex course after the procedure. Especially in the SAVR group, prolonged hospitalization rendered the 30-day mortality ineffective in describing short-term outcomes in this special population, as half of the patients stayed in hospital for more than 33 days. In fact, in-hospital mortality reached 25% in the SAVR group and the more favorable 30-day mortality of 10% masks the true outcome. However, short-term mortality in our cohort was lower after TAVI than after SAVR. Moreover, long-term follow-up was comparable in both groups, suggesting TAVI as a promising alternative therapy in this population.

The essential need for immunosuppression after kidney transplantation increases perioperative risk, especially the risk of wound healing deficits, and infectious complications, such as wound infections, pneumonia, and sepsis. Previous reports of cardiac surgery in KTR reported infectious complication rates of up to 17.5% [24] with rates of sepsis of up to 6% [25, 26], pneumonia of 7% [25, 26], and death due to sepsis of 11% [24]. Infective endocarditis of prosthetic valves is a worrisome complication after both SAVR and TAVI procedures, especially in patients on maintenance immunosuppressive therapy. To date, there are limited data to indicate any advantage of TAVI or SAVR regarding the risk of infective endocarditis after the procedure [27]. However, one patient after SAVR suffered from endocarditis in our cohort. The need for re-sternotomy in KTR is reported to be up to 22.8% [28], which goes in line with the high rate seen in our cohort. Glucocorticoids and mycophenolate mofetil [10], as well as diabetes mellitus (present in half of the study population) are known risk factors for wound healing deficits which might explain the high rates of infections and re-sternotomy in this population. We observed a reduced rate of severe complications, especially infections and wound healing deficits, after TAVI in our study. This probably resulted in shorter hospital stay and reduced in-hospital mortality. The majority of complications after TAVI in our study were associated with the vascular access. In the meantime, however, the size of the sheath for TAVI has been significantly reduced, and it is therefore suggested that this might translate into a lower vascular complication rates, as already demonstrated in other populations [29].

Following septic AKI, cardiac surgery-associated AKI (CSA-AKI) is the second most common type of AKI in adult patients undergoing open-heart surgery and is associated with increased mortality and morbidity [30]. Also, chronic kidney disease and AKI are the risk factors for an unfavorable outcome after TAVI [11–15, 31]. Additionally, pre-procedural kidney function is associated with new-onset dialysis [32], one-year mortality [33], and long-term progressing chronic kidney disease [34] after TAVI. A propensity-matched analysis of patients undergoing TAVI or SAVR showed a similar incidence of AKI in both groups. However, KTR were not included in this analysis [35]. More recently, Hundemer et al. [36] demonstrated a higher risk of AKI in KTR undergoing cardiac surgery compared to a matched control of non-transplanted patients. Another study found that the rate of AKI and AKI requiring dialysis was significantly lower after TAVI compared to SAVR [37]. Severe AKI bears the risk of permanent graft failure in KTR, which dramatically affects the quality of life. Despite comparable baseline graft function in our cohort, AKI was almost twice as frequent after SAVR compared to the TAVI group. Moreover, graft function recovered in all patients after TAVI, even if dialysis was temporarily necessary. As recently shown, AKI after TAVR is frequent in KTR [19] but seems to be transient. Nevertheless, severity, as classified by AKIN criteria, and the need for dialysis were higher after SAVR in our cohort, leading to permanent graft loss during hospitalization in 25% of these patients and additional graft failures in two patients during follow-up. Unfortunately, the fate of kidney grafts after cardiac surgery is rarely investigated, with small studies indicating that up to 15% of patients experience permanent graft loss after CSA-AKI [24, 26]. A recent multicenter registry study reported that 26.4% of KTR undergoing TAVI required long-term dialysis during follow-up [20]. Interestingly, in this study, only 1 out of 14 KTR recovered from peri-interventional hemodialysis and 6 KTR experienced graft loss during the follow-up. This rate is higher than those seen in our cohort and might be due to the fact that the patients in this registry were markedly older and included a considerable number of patients with stage 4 and 5 chronic kidney disease (15.3% and 22.2%, respectively) [20]. However, KTR should be meticulously informed about the risk of graft loss, including the risk of returning to dialysis, which, according to our data, might be more prevalent after SAVR.

### Limitations

There are some limitations of our study to discuss. First, because of the small sample size collected only in a single center, as well as the retrospective nature of the analysis, our study is susceptible to several biases that are common for this type of study such as allocation bias. Moreover, the small sample size further limits the validity of our results. Although each case has been discussed within the heart team on an individual basis including all available factors, we cannot exclude the risk of allocation bias. However, decision making toward the treatment concept for KTR with severe AS is complex and sometimes challenging. Our results are in favor of TAVI with regard to patient and graft survival, especially in the short- to mid-term follow-up, but are rather hypothesis-generating with respect to graft survival. Second, it has to be noted that the surgical group consisted of both, isolated aortic valve replacement and combined heart surgery, which thereby may have affected outcomes after SAVR. However, we found no numerical difference in complication rates between the two subgroups of surgically treated patients, especially with respect to renal outcomes. Despite the lack of randomized studies in this very special population, careful patient selection is mandatory, especially if combined surgery is discussed. Without a doubt, our study is mainly hypothesis-generating and further clinical studies are necessary to prove these results. In our opinion, our data may help clinicians to be aware, that KTR represent a special population that should be handled with upmost care.

## Conclusion

In summary, despite low peri-operative risk stratification in our cohort, in-hospital morbidity and mortality is high in KTR undergoing either TAVI or SAVR. Therefore, KTR are a high-risk population and treatment decision should be based on an individual basis with TAVI as a promising alternative for patient and graft survival in selected patients. Our data might help to fully inform KTR about the potential risks and benefits of both treatment strategies, although larger scaled studies are not available and would be difficult to conduct in this niche indication. TAVI is feasible and safe in KTR and might be an alternative to SAVR in patients with high-grade aortic stenosis after renal transplantation. In addition to a good short- to mid-term patient and graft survival, good long-term outcome in our cohort seems to be comparable to SAVR. Especially important for patients with a functioning graft is the fact that, compared to surgical valve replacement, the rate of graft failure and even graft loss is less likely to occur after TAVI. However, careful treatment planning and risk assessment in KTR with high-grade AS remain essential and more clinical data about this rarely investigated cohort are necessary.

**Conflicts of interest:** SB, CZ, AG, SP, HW, MR, HG, AMZ, and IAH state that they have no competing interests regarding this work. SF and TW are proctors for Edwards Lifesciences. SF, TW, and MVN are proctors for Abbott vascular.

**Funding:** Authors received no specific funding for this work.

## References

[1] Marwick TH, Amann K, Bangalore S (2019). Chronic kidney disease and valvular heart disease: conclusions from a Kidney Disease: Improving Global Outcomes (KDIGO) Controversies Conference.. Kidney Int.

[2] Raggi P, Boulay A, Chasan-Taber S (2002). Cardiac calcification in adult hemodialysis patients. A link between end-stage renal disease and cardiovascular disease?. J Am Coll Cardiol.

[3] Straumann E, Meyer B, Misteli M, Blumberg A, Jenzer HR (1992). Aortic and mitral valve disease in patients with end stage renal failure on long-term haemodialysis.. Br Heart J.

[4] Baumgartner H, Falk V, Bax JJ, De Bonis M (2017). ESC Scientific Document Group. 2017 ESC/EACTS Guidelines for the management of valvular heart disease.. Eur Heart J.

[5] Sharma A, Gilbertson DT, Herzog CA (2010). Survival of kidney transplantation patients in the United States after cardiac valve replacement.. Circulation.

[6] Smith CR, Leon MB, Mack MJ (2011). Transcatheter versus surgical aortic-valve replacement in high-risk patients.. N Engl J Med.

[7] Reardon MJ, Van Mieghem NM, Popma JJ (2017). Surgical or transcatheter aortic-valve replacement in intermediate-risk patients.. N Engl J Med.

[8] Ohno Y, Attizzani GF, Barbanti M (2015). Transcatheter aortic valve replacement for severe aortic stenosis patients undergoing chronic dialysis.. J Am Coll Cardiol.

[9] Büttner S, Weiler H, Zöller C (2016). Aortic valve stenosis in a dialysis patient waitlisted for kidney transplantation.. Ann Thorac Surg.

[10] Bootun R (2013 Feb). Effects of immunosuppressive therapy on wound healing.. Int Wound J.

[11] D’Errigo P, Moretti C, D’Ascenzo F (2016). OBSERVANT Research Group. Transcatheter aortic valve implantation versus surgical aortic valve replacement for severe aortic stenosis in patients with chronic kidney disease stages 3b to 5.. Ann Thorac Surg.

[12] Lüders F, Kaier K, Kaleschke G (2017). Association of CKD with outcomes among patients undergoing transcatheter aortic valve implantation.. Clin J Am Soc Nephrol.

[13] Gargiulo G, Capodanno D, Sannino A (2015). Moderate and severe preoperative chronic kidney disease worsen clinical outcomes after transcatheter aortic valve implantation: meta-analysis of 4992 patients.. Circ Cardiovasc Interv.

[14] Conrotto F, Salizzoni S, Andreis A (2017). Transcatheter aortic valve implantation in patients with advanced chronic kidney disease.. Am J Cardiol.

[15] Oguri A, Yamamoto M, Mouillet G (2015). FRANCE 2 Registry investigators. Impact of chronic kidney disease on the outcomes of transcatheter aortic valve implantation: results from the FRANCE 2 registry.. EuroIntervention.

[16] Bagur R, Webb JG, Nietlispach F (2010). Acute kidney injury following transcatheter aortic valve implantation: predictive factors, prognostic value, and comparison with surgical aortic valve replacement.. EurHeart.

[17] Sinning JM, Ghanem A, Steinhäuser H (2010). Renal function as predictor of mortality in patients after percutaneous transcatheter aortic valve implantation.. JaCCCardiovasc Interv.

[18] Fox H, Büttner S, Hemmann K (2013). Transcatheter aortic valve implantation improves outcome compared to open-heart surgery in kidney transplant recipients requiring aortic valve replacement.. J Cardiol.

[19] Al-Rashid F, Bienholz A, Hildebrandt HA (2017). Transfemoral transcatheter aortic valve implantation in patients with end-stage renal disease and kidney transplant recipients.. Sci Rep.

[20] Witberg G, Shamkhi J, Van Mieghem NM (2019 Sep). Transcatheter aortic valve replacement outcomes in patients with native vs transplanted kidneys: data from an international multicenter registry.. Can J Cardiol..

[21] Chawla LS, Bellomo R, Bihorac A (2017). Acute kidney disease and renal recovery: consensus report of the Acute Disease Quality Initiative (ADQI) 16 Workgroup.. Nat Rev Nephrol.

[22] Kappetein AP, Head SJ, Généreux P (2012). Valve Academic Research Consortium (VARC)-2. Updated standardized endpoint definitions for transcatheter aortic valve implantation: the Valve Academic Research Consortium-2 consensus document (VARC-2).. Eur J Cardiothorac Surg.

[23] Abdelfattah OW, Saad AM, Abushouk A (2021). Short-term outcomes of transcatheter aortic valve implantation versus surgical aortic valve replacement in kidney transplant recipients (from the US Nationwide Representative study).. Am J Cardiol.

[24] Zhang L, Garcia JM, Hill PC, Haile E, Light JA, Corso PJ (2006). Cardiac surgery in renal transplant recipients: experience from Washington Hospital Center.. Ann Thorac Surg.

[25] Musci M, Ankah CA, Klose H (2007). Heart valve operations in solid organ recipients: an 18-year single-center experience.. Transplantation.

[26] Basic-Jukic N, Ivanac-Jankovic R, Biocina B, Kes P (2015). Cardiovascular surgery after renal transplantation – indications, complications and outcome.. Ren Fail.

[27] De Palo M, Scicchitano P, Malvidini PG, Paparella D (2021). Endocarditis in patients with aortic valve prosthesis: comparison between surgical and transcatheter prosthesis.. Antibiotics (Basel).

[28] Rocha RV, Zaldonis D, Badhwar V (2014). Long-term patient and allograft outcomes of renal transplant recipients undergoing cardiac surgery.. J Thorac Cardiovasc Surg.

[29] Barbanti M, Binder RK, Freeman M (2013). Impact of low-profile sheaths on vascular complications during transfemoral transcatheter aortic valve replacement.. EuroIntervention.

[30] Nadim MK, Forni LG, Bihorac A (2018). Cardiac and vascular surgery-associated acute kidney injury: the 20th international consensus conference of the ADQI (Acute Disease Quality Initiative) Group.. J Am Heart Assoc.

[31] Gupta T, Goel K, Kolte D (2017). Association of chronic kidney disease with in-hospital outcomes of transcatheter aortic valve replacement.. JACC Cardiovasc Interv.

[32] Hansen JW, Foy A, Yadav P (2017). Death and dialysis after transcatheter aortic valve replacement: an analysis of the STS/ACC TVT registry.. JACC Cardiovasc Interv.

[33] Levi A, Codner P, Masalha A (2017). Predictors of 1-year mortality after transcatheter aortic valve implantation in patients with and without advanced chronic kidney disease.. Am J Cardiol.

[34] Haase-Fielitz A, Altendeitering F, Iwers R (2020). Acute kidney injury may impede results after transcatheter aortic valve implantation. Clin.. Kidney J.

[35] Thongprayoon C, Cheungpasitporn W, Srivali N (2015). AKI after transcatheter or surgical aortic valve replacement.. J Am Soc Nephrol.

[36] Hundemer GL, Srivastava A, Jacob KA (2021). Acute kidney injury in renal transplant recipients undergoing cardiac surgery.. Nephrol Dial Transplant.

[37] Kumar N, Garg N (2019). Acute kidney injury after aortic valve replacement in a nationally representative cohort in the USA.. Nephrol Dial Transplant.

